# The Immune and Regenerative Response to Burn Injury

**DOI:** 10.3390/cells11193073

**Published:** 2022-09-29

**Authors:** Matthew Burgess, Franklin Valdera, David Varon, Esko Kankuri, Kristo Nuutila

**Affiliations:** 1 United States Army Institute of Surgical Research, Fort Sam Houston, TX 78234, USA; 2 Department of Pharmacology, Faculty of Medicine, University of Helsinki, 00014 Helsinki, Finland

**Keywords:** burn injury, immune response, inflammation, tissue regeneration

## Abstract

Burn are diverse and complex injuries that not only have local effects but also serious systemic consequences through severe and prolonged inflammatory response. They are caused by heat, electricity, friction, chemicals, or radiation and are commonly divided into superficial, superficial partial-, deep partial- and full-thickness injuries. The severity of the burn depends mainly on the size and depth of the injury but also on location, age, and underlying systemic diseases. A prolonged and strong immune response makes major burns even worse by causing multiple systemic effects including damage to the heart, lungs, blood vessels, kidneys, and other organs. Burns that do not require surgical excision, superficial and superficial partial-thickness, follow the known progression of wound healing (inflammation, proliferation, remodeling), whilst deep partial- and full thickness injuries requiring excision and grafting do not. For these burns, intervention is required for optimal coverage, function, and cosmesis. Annually millions of people worldwide suffer from burns associated with high morbidity and mortality. Fortunately, over the past decades, burn care has significantly improved. The improvement in understanding the pathophysiology of burn injury and burn wound progression has led to developments in skin grafting, fluid resuscitation, infection control and nutrition This review article focuses on the immune and regenerative responses following burn injury. In the Introduction, we describe the epidemiology of burns and burn pathophysiology. The focus of the following chapter is on systemic responses to burn injury. Next, we define the immune response to burns introducing all the different cell types involved. Subsequently, we discuss the regenerative cell response to burns as well as some of the emerging novel treatments in the battle against burns.

## 1. Introduction

According to the World Health Organization (WHO), burns are a global health problem [1]. These injuries are painful and devastating, and they are associated with high morbidity and mortality. Millions of people worldwide suffer from burns each year, many require hospitalization and close to 200,000 die. Annually in the US, half a million burn injuries require medical attention and approximately 40,000 people are hospitalized. These patients also suffer high percentages of mortality, making burns the leading cause of unintentional death in the US (Burn Incidence fact sheet). Burns are even a bigger burden in low- and middle-income countries where more than 90% of the burn deaths occur [2,3]. Fortunately, over the past decades, burn care has significantly improved. The improvement in understanding the pathophysiology of burn injury and burn wound progression has led to developments in skin grafting, fluid resuscitation, infection control and nutrition [4]. Today, patients suffering from a 90% total body surface area (TBSA) burn can survive [5]. However, burn injury survivors often suffer from long-lasting physical disablements, emotional distress, and decreased quality of life [6].

**Figure 1:**
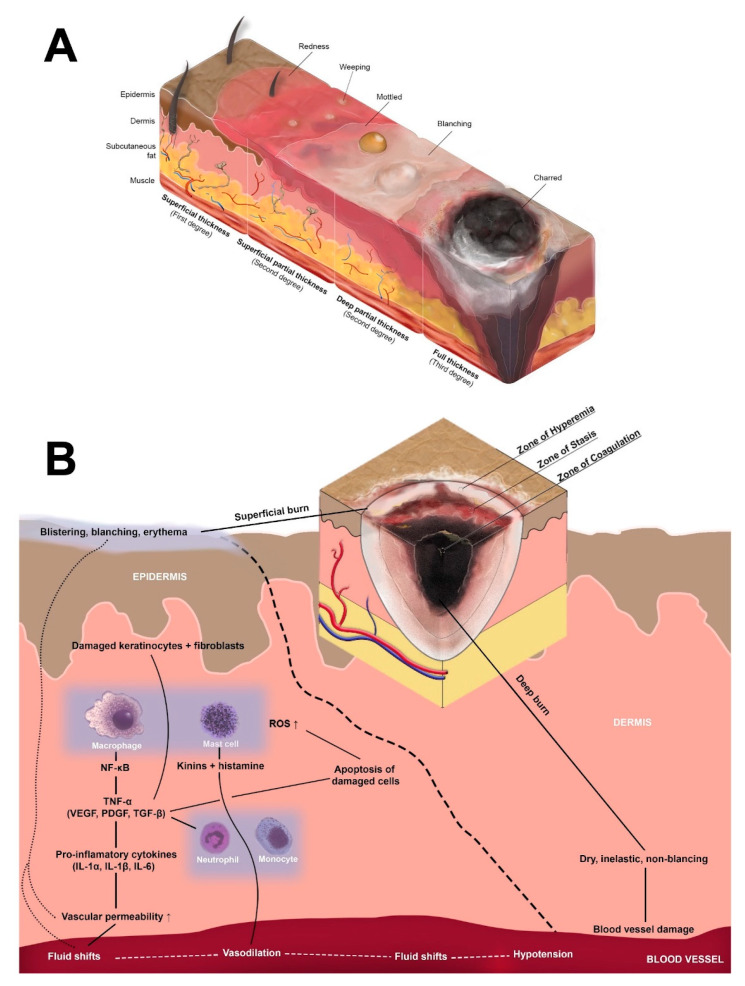
(**A**) Burn depth. Burns are classified as superficial, partial thickness—superficial or deep, or full thickness. Superficial burns damage the different layers of skin, while deep burns damage soft (tissue fat and muscle) and even bone. (**B**) Jackson’s Zones. Burns consist of three distinguished zones of coagulation, stasis and hyperaemia. The zone of coagulation is the innermost zone, the primary site of the injury and once the burn occurs its cells will rapidly undergo necrosis. The surrounding zone of stasis is characterized by tissue damage and ischemia but may still be potentially salvageable. The outermost zone of hyperaemia will usually recover but is characterized by substantial local swelling and redness caused by the immediate inflammatory response to the injury. The innate immune system and its cells are the body’s first line of defense against invading pathogens following burn injury. Characterized by defense against pathogens and disposal of necrotic tissues, the innate immune response paves the way for the proliferative and remodeling phases of wound healing following a burn injury. The picture depicts local inflammatory response to superficial burn in the dermis.

A burn is tissue damage caused by heat, electricity, friction, chemicals or radiation. The classification of burns usually depends on the depth of the injury and, thus they have been categorized into superficial, superficial partial-, deep partial- and full-thickness injuries. Superficial burns damage the different layers of skin, while deep burns damage soft (tissue fat and muscle) and even bone [4]. The severity of the burn depends mainly on the size, depth, and location of the injury, as well as on age and underlying systemic diseases. Minor burns, affecting less than 10% TBSA, usually heal well, whereas major burns, categorized to affect more than 20% TBSA, often require surgical treatment to heal. These larger burns are also more prone to complications, such as infections, sepsis, and scarring of the skin (Figure 1A) [7].

Burns differ from other skin wounds by consisting of three distinguished zones of coagulation, stasis, and hyperemia. The zone of coagulation is the innermost zone, the primary site of the injury and once the burn occurs its cells will rapidly undergo necrosis [8]. The surrounding zone of stasis is characterized by tissue damage and ischemia but may still be potentially salvageable. The outermost zone of hyperemia will usually recover but is characterized by substantial local swelling and redness caused by the immediate inflammatory response to the injury [9]. Once activated, the inflammatory reaction stimulates the innate immune response and its cells to protect the injured area from pathogens. Furthermore, inflammatory cells remove dead cells and debris, paving the way for keratinocytes and fibroblasts to initiate tissue repair (Figure 1B) [10].

However, when it comes to severe burn injuries, the immune response is prolonged and strong, making the injury even worse by causing multiple systemic effects, and damaging several organs such as the heart, lungs, blood vessels, and kidneys [11]. In severe burns, the heat damage increases the permeability of the capillaries, inducing fluid loss and allowing plasma to leak out of circulation. In addition, fluid can become trapped inside the body, causing edema. The rapid loss of fluid may lead to burn shock, described as decreased cardiac output, increased vascular resistance, hypovolemia, and hypo-perfusion [12]. A prolonged and severe inflammatory reaction can also lead to multiple organ failure due to systemic inflammatory response syndrome (SIRS) that is marked by fast heart rate, low blood pressure, low or high body temperature, and low or high white blood cell count [13]. Furthermore, prolonged inflammation at the burn site can cause excessive growth of granulation tissue and wound contraction, which ultimately leads to scarring which can be disfiguring, functionally restrictive and may require revisionary surgeries [14].

The purpose of this review is to describe the immune and cell responses to burn injury. Therefore, the first chapter describes the systemic responses to burn injuries. The following chapter is focusing on the immune response to burns, introducing all the different cell types involved. Subsequently, the regenerative cell response to burns is explained as well as strategies to mitigate severe inflammatory response post-burn. “Finally, the discussion will address the importance of research and approaching novel treatments in the battle against burns.

## 2. Systemic Response to Burns

The impact of burns may spread beyond the areas of direct injury. Indeed, severe burns, regardless of origin, trigger a systemic inflammatory response. The effects exhibited within each organ system are both the result of the burn injury, as well as the response from that organ system to it. The threshold for eliciting a systemic response is estimated at 20% or more of TBSA burned severely—beyond superficial depth. Multiple organ systems are involved, with an emphasis on the circulatory system causing hemodynamic shifts, the respiratory system with increased respiratory rates or work of breathing, the endocrine system with hypermetabolism and hyperglycemic states, and alterations of frequency and function of the immune system. Systemic responses to burns range from a near-normal basal metabolic rate (BMR) at TBSA of <10%, increase with severity, until plateauing at nearly doubled normal BMR with a TBSA of 40% and beyond [15]. The following will discuss the effects of severe burns on these organ systems and the systemic impacts that follow.

### 2.1. Inflammatory Response

Burns initiate a massive inflammatory response. After a severe burn, inflammation is systemically increased throughout the body with an elevation of circulating cytokines such as tumor necrosis factor alpha (TNF-α) and interferon gamma (INF-γ). Systemic inflammatory response syndrome (SIRS) is a condition in which the immune system is up-regulated in response to a disruption of equilibrium in the cellular environment. The SIRS criteria—an elevation in heart rate, increased body temperature and respiratory rates, and a surge in circulating white blood cells—is a presentation common after burn injury, especially after severe burns that induce a systemic response. This effect on the body is directly proportional to the magnitude of the burn and exists on a spectrum. That is, the greater the depth of injury and the greater the surface area affected, the larger the systemic response [16].

### 2.2. Multi-Organ System Involvement

Systemic inflammation increases the permeability of capillaries, allowing extravasation of fluid into the interstitium, worsening peripheral edema, and exacerbating hypotension due to less volume remaining within vessels and the circulatory system. The heart rate and systemic vascular resistance are elevated to compensate for this phenomenon, as an attempt to maintain perfusion throughout the body. Respiratory function is affected by constriction of the bronchi, which leads to increased work of breathing. Tachypnea ensues, and this further contributes to energy utilization and elevation of metabolic demand. Despite increased peripheral immune cell abundance is increased due to sustained trauma from a severe burn, there is a tamponade of the immune response due to inhibition of cell-mediated and humoral protection. Signaling initiated by the damage-associated molecular patterns (DAMPs) or danger signals, such as DNA, ATP, high mobility group box 1 (HMGB1) or cytokines and other alarmins released from the dying cells can dysregulate inflammation and immune response control leading to hyperactivation or failed suppression of inflammatory signals [17,18,19,20,21]. Increased activity of suppressing DAMPs, also called SAMPs, such as prostaglandin E2 aimed at eventually resolving the inflammatory activation, can also render the immune system vulnerable [22]. This places those severely burned at an increased risk for infection beyond an already troublesome removal of the integument as a first line defense [23].

### 2.3. Hypermetabolism and Hyperglycemia

Hypermetabolism occurs in moderate and severe burns, and it increases proportionally to the depth and size of injury. Post-injury hypermetabolic states are manifested as dynamic responses in an upregulation of hormones, activity of the circulatory system, and the immune response, although less protective. BMRs are elevated following severe burns and can even surpass twice normal [24]. Catecholamine release elevates body temperature, heart rate, and blood pressure. Hypermetabolic states are associated with increased glucocorticoids, which induce catabolism of circulating proteins and lipids. Because of this, a rapid turnover of metabolic building blocks quickly exhausts their supply, exacerbating the catabolic state. Resources are endogenously generated to facilitate these hypermetabolic pathways—protein synthesis, lipogenesis, gluconeogenesis, hormone production, etc. Muscle wasting is a sequela of this catabolic phenomenon, as the scarcity of amino acids coupled with excessive demand subsequently promotes the breakdown of protein in muscle to provide them. Treatment should focus on the attenuation of hypermetabolism, as this state can last up to a year. Hyperglycemia is a known consequence of severe burns, and has multiple causes, including elevated rates of gluconeogenesis and glycogenolysis, due to hypermetabolism, and an increase in cellular insulin resistance. Insulin resistance worsens the hyperglycemic state due to a decreased utilization of circulating glucose in abundance. This underutilization exacerbates the breakdown of proteins and lipids through the atrophy of muscle, to instead be used as an energy source given the impairment of glucose metabolism. Hyperglycemic states impair wound healing by dampening the immunologic response, which leads to a vulnerable, immunosuppressed state [25,26].

### 2.4. Burn Shock

When the systemic response of severe burns and involvement of multiple organ systems manifests as a malperfused state, burn shock can ensue. When organs lack perfusion to a degree such that dysfunction and cellular death occur, this is termed shock. There are various causes of shock, for example, sepsis from an infectious source, which can progress to organ dysfunction. However, burn shock is unique in that it is characterized by a depression of activity in the myocardium, in addition to an increase of vascular permeability, resulting in a two-fold reduction of circulating volume and thus perfusion. A decrease in myocardial activity circulates less blood, oxygen, and nutrients. Fluid shifts occur in tandem through increased vascular permeability, as previously mentioned. The oncotic pressures rise in the interstitium and connective tissue, which causes fluid to follow, leading to the collapse of peripheral vasculature. Ultimately, these issues can lead to malperfusion and end-organ ischemia, which requires aggressive resuscitation so that circulation is maintained, and delivery of blood, immune cells, nutrients, cytokines and growth factors where they are required can help with response to the insult [27].

### 2.5. Sepsis

Burn wound infections are common and may often result in invasive infections. The larger the surface area and the deeper the burn, the greater the risk is to develop a life-threatening invasive infection that can progress to sepsis. Sepsis is the body’s ultimate response to infection and the leading cause of death in severe burn patients. It is defined as “severe organ dysfunction attributed to host’s disordered response to infection”. Typically sepsis is caused by bacterial infections, but it can also be caused by fungi and viruses. Due to skin loss, sepsis in burn patients is distinguished from general sepsis since the risk of infection is present as long as the injured areas are unhealed [28,29]. In the pathophysiology of sepsis, the body’s immune system, releases endogenous DAMPs and exogenous pathogen-derived molecular patterns (PAMPs) that activate Toll-like receptors (TLR) on the surface of antigen-presenting cells and monocytes. This triggers widespread inflammation, initiating the clinical syndrome of sepsis that causes continuing tissue damage ultimately leading to multi-organ dysfunction. Septic shock is the final, most severe form of sepsis in which blood pressure drops to an alarming low level. Most burn-related deaths in modern burn units occur because of septic shock [30,31].

## 3. The Innate Immune Response to Burns

**Figure 2:**
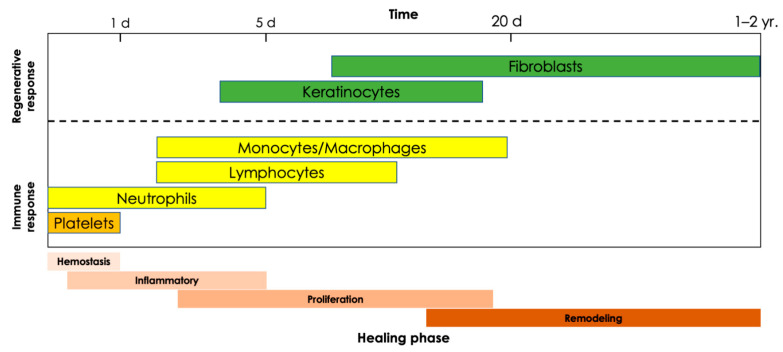
Summary of the main immune and regenerative cells involved in burn wound healing at different stages of healing. Abbreviations: d, day; yr, year.

The innate immune system and its cells are the body’s first line of defense against invading pathogens following burn injury. Characterized by defense against pathogens and disposal of necrotic tissues, the innate immune response paves the way for the proliferative and remodeling phases of wound healing following a burn injury (Figure 2). The cells of the innate immune system include neutrophils, monocytes and macrophages, natural killer cells, dendritic cells, and mast cells, which are collectively responsible for the acute inflammatory response to thermal injuries. While some of these cells are resident dermal cells adjacent to the site of injury, most immune cells are recruited from circulation and the surrounding vasculature [32]. Within hours of the initial injury, the degranulation of platelets releases signals, including cytokines, chemokines, and microRNAs, which recruit leukocytes to the site of injury before activating and modulating their functions [33]. Upon arrival, leukocytes, namely neutrophils, monocytes and macrophages, and mast cells, release numerous additional growth factors and signaling proteins responsible for spatial and timeline-appropriate modulation of proliferation and differentiation as required for progression of the wound healing process from the hemostasis and inflammatory phases into the proliferation and remodeling phases (Table 1) [34]. These cells and processes represent the cell-mediated aspect of the innate immune response. In contrast, the innate immune system exerts humoral immunity through complement activation with the goal of targeted pathogen destruction, which it achieves through three pathways: the classical, lectin, and alternative pathways. The classical pathway uses antigen-antibody complexes, the lectin pathway targets pathogens with mannose, and the alternative pathway is involved in targeting pathogens in injured tissues. Notably, all three pathways intersect at the C3 complex and culminate in the formation of membrane attack complexes capable of targeted pathogen lysis. In the context of burn wounds, excessive complement activation results in a prolonged inflammatory response. Animal burn models have shown increases in complement levels, namely complexes C3, C3a, and C5a, which are products of all three pathways. Both C3a and C5a have been shown to have hemostatic, antibacterial, and pro-inflammatory effects, which are important to wound healing, yet counterintuitive when activated in excess (Figure 1B) [35].

### 3.1. Neutrophils

Neutrophils are the first leukocytes to respond to tissue damage and are recruited from the surrounding microvasculature via up regulation of P-selectin on the surface of vascular endothelial cells and circulating platelets. P-selectin binds to the surface of neutrophils, allowing them to tether and roll along the vascular endothelium before undergoing diapedesis and entering the inflamed tissues [36]. Upon arrival, neutrophils serve three purposes: microbe elimination, extracellular matrix (ECM) clearance, and recruitment of additional inflammatory cells (Table 1) [37,38].

Neutrophils use phagolysosomes, digestive vesicles formed by the fusion of a phagosome and a lysosome, to neutralize bacteria, resulting in the subsequent release of free radicals, such as reactive oxygen species (ROS), into the environment, which tend to also damage otherwise healthy cells near the site of injury [13,38]. Neutrophils have also been shown to kill invading microbes by trapping them in extruded extracellular nets of histones and DNA [39]. This programmed process called NETosis, frequently leading to cell death, can be activated by microbes or their components as well as by cytokines and chemokines. Interestingly, formation of extracellular traps is not a unique feature of neutrophils as also basophils, eosinophils and mast cells as well as monocytes and macrophages can undergo a similar type of programmed response [40,41,42,43]. Neutrophils are additionally responsible for clearing damaged ECM via the production of enzymes such as matrix metalloproteinase (MMP), elastase, and collagenase clearing the way for the formation of new ECM down the road [38]. Lastly, neutrophils release numerous cytokines, including TNF-α, interleukin(IL)-1 beta (IL-1β), and IL-6, which further promote inflammation and recruit additional neutrophils, monocytes, and macrophages to the site of injury, thereby perpetuating the innate immune system’s response [38]. The impact of burns on the neutrophilic component of wound healing include impedance of migration speeds during the first 5 days post-injury, prolonged infiltration of neutrophils (and macrophages) for weeks after injury, as well as impaired chemotaxis, phagocytic function, and bactericidal capacity [44,45,46]. However, a series of experiments in guinea pigs from the 1970s demonstrated that the elimination of neutrophils from the wound healing process did not significantly perturb tissue repair [47]. More recently, neutrophil knockdown experiments in diabetic mice support these findings and go on to report that the progression of wound healing is in fact more rapid in the absence of neutrophils [48]. These studies suggest that the role of the neutrophil may not be essential to the innate immune system’s inflammatory response to injury, in so much that the functions carried out by neutrophils may be sufficiently carried out by other leukocytes responding to the injury without a detrimental impediment to the overall wound healing process. While these findings are meant to highlight the specific role of neutrophils and the overlap of duties between different leukocytes, it is worth illuminating the potential oversight of this theory in that rapid wound healing does not necessarily equate to effective or optimal wound healing (Figure 2 and Table 1).

### 3.2. Monocytes and Macrophages

Monocytes are the precursors to macrophages and both populations of phagocytes play important roles in immune function regulation, clearance of cellular debris, and tissue repair [49,50]. Following their activation, these leukocytes transiently maintain their morphology as monocytes while secreting pro-inflammatory and angiogenic factors (e.g., IL-8, IL-1β, and TNF-α) before differentiating into macrophages [51,52]. This differentiation process is regulated, in part, by microRNAs such as miR-146a which modulates polarization of macrophages between the M1 and M2 phenotypes to balance pro- and anti-inflammatory operations (Figure 2 and Table 1) [53].

Following differentiation, macrophages serve as phagocytes to clear the burn wound of damaged matrix and cellular debris, including spent neutrophils and platelets [13,52]. Initially presenting with the pro-inflammatory or M1 phenotype, macrophages eventually transition to an anti-inflammatory or M2 phenotype [50]. M1 cells are characterized as a source of pro-inflammatory mediators such as prostaglandin E2, reactive oxygen and nitrogen intermediates, TNF-α, IL-1, IL-6, and IL-8 [38,49]. In contrast, the M2 phenotype aims to resolve inflammation while simultaneously promoting angiogenesis and resolution within the healing cascade by producing factors such as the IL-1 receptor antagonist, PDGF, TGF-β1, and FGF which promote ECM production and angiogenesis [34,38,49] (Table 1). Several signals govern or contribute to the macrophage polarization status. Such important cues are the cells’ microenvironmental factors (oxygen concentration, pH, etc.) and the availability of substrates for metabolism [54,55]. Given the important regulatory role of the macrophage as a gatekeeper for providing an environment supportive or destructive for tissue healing, it is likely that more categorical subtypes on the macrophage spectrum are identified as more information on the regulation of the dynamic nature of macrophage responsivity emerges [55,56].

### 3.3. Mast Cells

Mast cells are immune cells that reside in the dermal layer of the skin, promote acute inflammation, stimulate re-epithelialization and angiogenesis, and have been shown to play an important role in skin scarring [57]. Thermal injuries cause the degranulation of resident mast cells which in turn results in the release of histamine, TNFα, prostaglandins, IL-1, and IL-6 [21,58]. These cytokines lead to increased vascular permeability thereby promoting and aiding in the recruitment of neutrophils and monocytes to the site of injury [59,60]. The release of histamine ultimately leads to an uptick in the production of reactive oxygen species (ROS) and triggers a rapid exocytosis of P-selectin that is a protein produced by activated platelets and endothelial cells [61]. Similarly, the release of TNF-α by mast cells upregulates the expression of E-Selectin, a receptor produced by endothelial cells. Both P- and E-Selectin are responsible for improved adhesion of leukocytes to the luminal surface of the vascular endothelial cells, thereby aiding in migration to the site of injury [36]. A knockdown experiment of these selectins reports significantly impaired wound closure with suppressed keratinocyte migration, angiogenesis, granulation tissue formation, leukocyte infiltration, and expression of transforming growth factor beta (TGF-β) and IL-6 [62]. This indicates the importance of mast cell signaling in the effective recruitment and migration of additional immune cells to the site of injury.

Notably, mast cells are not only present during the early phases of the immune system’s response to burn injuries but are also recruited to the wound site during the latter stages of wound healing to aid in ECM remodeling through the production of tissue plasminogen activator and plasminogen activator inhibitor-1 [63]. Mast cells have also been shown to release IL-10 to dampen an excessive immune response, as well as proteases, like chymase, which leads to fibroblast recruitment via conversion of TGF-β1 and MMP-9 to their active forms [63,64]. Along the same thread, mast cells release vascular endothelial growth factors (VEGFs), fibroblast growth factors (FGFs), and plateled-derived growth factor (PDGF) which aid in preparation of the injury site for the repair phase of wound healing (Table 1) [63].

### 3.4. Natural Killer (NK) and Dendritic Cells

Natural killer (NK) cells are innate lymphocytes of the dermis important to pathogen destruction and the early immune response. NK cells have a reciprocally regulatory relationship with dendritic cells resulting in a unique and potent crosstalk of activation responsible for antibacterial defenses [65]. NK cells are activated by type I & III interferons (IFN) and promote the synthesis of Type II IFN, IFN-γ, and TNF-α following their activation [66]. Upon their arrival to the site of injury, NK cells induce programed cell death in infected cells via cytolytic activity characterized by the release of cytotoxic granules [67]. A study of a murine burn injury model found decreased levels of IL-12 and CCL3 production by NK cells following full-thickness burns later noting increased levels of these cytokines in partial thickness burns compared to healthy controls [68]. CCL3 is an important modulator of monocyte and neutrophil recruitment while IL-12 prompts the release of IFNγ from dendritic cells and activates additional NK cells [69,70]. This seems to indicate that full thickness burns result in dysfunctional leukocyte recruitment from the perspective of the NK cell (Table 1).

Dendritic cells are phagocytic and antigen-presenting dermal leukocytes which serve as “immune sentinels” for T-cells while also augmenting the early immune response and clearing cellular debris [71]. It has been proposed that dendritic cells sense dermal injuries via host-derived nucleic acids via Toll-like receptors 7 and 9 and that this is the process through which they are recruited to the site of injury [72]. Like macrophages, dendritic cells are responsible for phagocytosis of damaged tissue at the injury site. They have also been shown to play a role in wound repair [73]. As previously mentioned, dendritic and NK cells have a reciprocal activation relationship which is achieved through the production of type I interferons by dendritic cells, which leads to the activation of NK cells [66,72]. The significance of the dendritic cell in the wound healing process is highlighted by a study in mice which found delayed burn wound closure in dendritic cell deficient mice. This impaired wound healing was associated with significant suppression of early cell proliferation, formation of granulation tissue, and TGF-β1 levels as well as angiogenesis (Table 1) [73].

## 4. The Adaptive Immune Response to Burn

The adaptive immune system or the acquired immune system consists of leukocytes, called lymphocytes, which can further be classified as T-cells and B-cells. Responsible for identification and elimination of foreign invaders, known as antigens, the adaptive immune system’s response to burn injury is largely dedicated to infection control. The adaptive immune system responds to these antigens in two ways: the cell-mediated and humoral immune responses carried out by T- and B-cells, respectively. The cell-mediated immune response involves the activation of T-cells which target host cells which have already been infected with an antigen by directly interacting with them before activating macrophages to destroy the invading microbe. The humoral response, however, uses B-cells, which secrete antibodies, also known as immunoglobulins, which then bind to the foreign antigen, thereby blocking their ability to bind to receptors on host cells and infect them. These antibodies additionally serve as markers for phagocytic cells of the innate immune system, labeling these antigens for destruction. Following a full thickness burn injury, which compromises the dermis’ integrity as a protective barrier, the adaptive immune response aids wound healing by eliminating any invading pathogens, while simultaneously modulating the innate immune response to the injury (Figure 1B) [74].

### 4.1. T-Cells

T-cells are the primary lymphocyte of the cell-mediated immune response; they are responsible for augmentation of the innate immune system and direct defense against foreign antigens. Compared to most other tissues, healthy skin and epithelium are unique in that they have a predominately Gamma-Delta (γδ) T-cell subpopulation, which has been shown to be pro-regenerative and plays a key role in regulation of infiltration, inflammation, and healing of burn wounds [75,76,77,78]. Data from a murine burn model found increased levels of γδ T-cells in circulation early after injury additionally noting their importance as a source of chemokines [79]. The importance of this subpopulation of T-cells was again highlighted by the observation of a high mortality rate during the first 48 h post-injury in mice deficient in γδ T-cells [80]. Furthermore, it has been shown that this subset of T-cells regulates the infiltration of both myeloid cells and Alpha-Beta (αß) T-cells to the wound site during the acute stages of wound healing, before aiding in the transition from the inflammatory phase to the proliferative phase of wound healing [81]. A second study in murine burn wound models supported these findings, with data indicative of high levels of αβ T-cell infiltration to the wound site following thermal injuries. The same study notes that this T-cell phenotype suppresses lymphocyte proliferation while producing pro-inflammatory cytokines, such as IL-17, a cytokine key to linking T-cell activation to neutrophil mobilization and activation [24]. Therefore, despite healthy dermal tissues containing mostly γδ T-cells, it is their recruitment of αβ T-cells following thermal injuries that makes them the dominating subpopulation at the site of an injury, thereby favoring inflammation over proliferation during the early stages of wound healing (Table 1). There are other unconventional T cell subsets called Mucosal-associated invariant T (MAIT) cells and Invariant Natural Killer T (iNKT) cells that have been the subject of many recent studies. They trigger rapid immune responses, regardless of the major histocompatibility complex (MHC) expression and recent studies have shown that both cell types may have capacity to participate in tissue repair [82,83,84].

Naïve T-cells can differentiate into T helper 1 (Th1) or T helper 2 (Th2) cells. Th1 cells are generally associated with a pro-inflammatory state and secrete IL-2 and IFNγ following their activation and differentiation. The Th2 phenotype, on the other hand, secretes cytokines that promote apoptosis, and an anti-inflammatory response including IL-4, IL-5, and IL-10, which in turn prompts B-cells to produce antibodies. Moreover, several additional T-cell subsets have been identified. Their role in damage repair and in burns is under intensive investigations [24,85].

### 4.2. B-Cells

In addition to the aforementioned cytokines, IL-4 and IL-5, B-cells have been shown to initiate immunoglobulin or antibody production in response to IL-15 which is secreted by dendritic cells, monocytes and macrophages, and endothelial cells [86]. However, in the context of burn injuries, both local and systemic immunoglobulin or antibody levels have been shown to be significantly decreased during the first 48 h following burn injuries with serum levels remaining low for weeks in some patients [87,88]. This indicates that the role of B-cells may be down-regulated following thermal injuries, providing further evidence for the difficulty faced in infection control following burns (Table 1).

## 5. Regenerative Response to Burn Injury

In cases of full-thickness burns when both epidermis and dermis have been destroyed, surgical treatment is required since the burn wounds are not capable of regenerating on their own. The standard of care treatment is to excise the burnt tissue and replace it with an autograft [89]. Superficial and partial-thickness burns may heal without surgical intervention following the steps of normal wound healing. The repair of injured tissue overlaps with the tail end of the inflammatory phase, and keratinocytes and fibroblasts are the main cell types responsible for restoration of the burnt tissue. The proliferation phase, as its name implies, is characterized by cell proliferation with the goal of achieving wound closure. Wound closure is characterized by complete re-epithelization of the affected integument and is of upmost importance to restoring the most vital functions of the skin, including fluid regulation and protection from infections and UV damage [90]. Following the formation of granulation tissue within the wound site, fibroblasts secrete collagen, which forms the new extracellular matrix, while the overlying keratinocytes migrate to cover the wound site. The proliferative phase may begin as early as hours after injury in partial thickness burns. This eventually transitions into the remodeling phase [91] (Figure 2).

### 5.1. Keratinocytes

Keratinocytes are the predominant cell type in the epidermis and are responsible for the formation of a new epithelium, and restoration of the skin’s barrier function following a burn injury. Healthy, uninjured skin is maintained by keratinocytes which proliferate in the basal layer, differentiate as they migrate through the granular layer, and finally become the flattened dead cell remnants of the cornified layer [92]. In response to epidermal damage following a burn, a host of cytokines and growth factors secreted by the immune cells recruit and activate the uninjured keratinocytes which in turn prompts them to migrate across the wound space covering the epidermal defect created by the thermal injury [92,93]. Keratinocytes achieve this migration by first loosening their desmosome attachments to one another and hemidesmosome attachments to the local basal lamina allowing them the flexibility necessary for migration [94]. Growth factors (EGF, KGF, TGF-β, VEGF, and Fibroblast Growth Factors), cytokines (IL-1, IL-6, IL-8, and TNF-α), integrins, keratins, and matrix metalloproteinases (MMPs) released by fibroblasts in the underlying granulation tissue promote keratinocyte proliferation and migration across the wound [95,96] (Table 1). Keratinocytes have also been shown to work alongside fibroblasts and endothelial cells to promote angiogenesis during remodeling via the release of growth factors such as MSF, NGF, VEGF, and GM-CSF as well as aid in follicle and sweat gland regeneration [97]. Keratinocytes additionally play a role in immune regulation via cytokine signaling of immune cells releasing factors such as IL-8, IFN-β, and TNF-α (Table 1) [98,99].

### 5.2. Epidermal Stem Cells

Epidermal stem cells reside in the epidermal appendages (sebaceous glands, eccrine sweat glands, and hair follicles). These include the basal epithelial, eccrine gland, follicular, dermal papillary, and bulge cells [97]. In the context of wound healing, these stem cells go beyond their baseline function of self-renewal to differentiate and migrate to the wound space to aid in acute re-epithelialization in response to IL-1 and TNF-α signaling [99]. Upon arrival at the wound site, these cells release cytokeratins, basement membrane proteins, and growth factors such as epidermal growth factor (EGF), TGF-β, VEGF, and insulin-like growth factors (IGFs) with the goal of regenerating damaged or necrotic keratinocytes, melanocytes, hair follicles, and sweat glands following a burn injury (Table 1) [97].

### 5.3. Melanocytes

Melanocytes are rooted in the basal layer of the epidermis with dendrites extending into the spinous layer of the epidermis and are responsible for pigmentation of the skin and in turn prevention of UV damage to the skin [97]. Depigmentation is common in burn patients. Carney et al. (2021) suggested that depigmentation is caused by either hypo- and hyper-pigmentation are the result of hypo- or hyper-activity of melanocytes rather than the absence or abundance of this cell type in post-burn healing skin, respectively. In addition to their aforementioned roles, keratinocytes play a part in melanin synthesis by propagating damage-associated signals which are then received by melanocytes which in turn synthesize melanin from tyrosine. Melanin is then transferred back to the keratinocytes to protect them against nuclear damage [100]. However, burns result in dysregulation of this process, potentially through the “inactivation” of melanocytes. Carney et al. (2021) have proposed that these post-burn melanocytes can be reactivated using alpha melanocyte stimulating hormone (α-MSH), resulting in renewed pigmentation and an increased capacity to guard the new keratinocytes from UV damage (Table 1) [100].

### 5.4. Fibroblasts

Fibroblasts promote the formation of connective tissues during wound healing and dermal remodeling, while also aiding in re-epithelialization. Following the inflammatory phase of wound healing, fibroblasts migrate to the wound site and begin reformation of the extracellular matrix with collagen deposition. In the early phase of healing, the ratio of type III to type I collagen production is higher than that of a mature scar or normal skin [101]. As the wound progresses, balance is restored with type I collagen being most abundant. Most of these fibroblasts end up differentiating into myofibroblasts, most notable for their contractile phenotype and expression of alpha smooth muscle actin (α-SMA), which are responsible for wound contraction during the remodeling phase [102]. In an ideal wound healing setting, both fibroblasts and myofibroblasts carry out their duties prior to programmed cell death via apoptosis. However, dysregulation of this process leads to prolonged activation, which can lead to contractures and the formation of hypertrophic burn scars [103]. Long non-coding RNAs have been shown to be essential to this process influencing both the proliferation and apoptosis of dermal fibroblasts postburn [104,105]. Increased expression of these long 28 non-coding RNAs in fibroblasts promotes the autocrine secretion of TGF-β2 which leads to hypertrophic scar (HTS) and keloid formation [106]. Nevertheless, multiple therapies have been introduced and tested to alleviate the morbidity associated with HTS secondary to fibroblast activity (Table 1) [107].

## 6. Chronic Inflammation following Burn Injury

If the acute inflammatory response to a burn is prolonged, it may lead to excessive healing. Consequently, burn wounds heal by repair instead of regeneration, causing excessive growth of granulation tissue and wound contraction, which ultimately leads to formation of scar tissue [108]. Although hypertrophic scar formation can occur following any injury in etiology, it is of particular concern and focus subsequent to burns due to exacerbating the risks of impaired functional status and cosmesis. Specifically, contractures, chronic pain, pruritus, and unacceptable scar appearance. Chronic inflammation is the most important risk factor for hypertrophic scarring. An impaired inflammatory response leads to stagnation in the wound healing cascade at variable intervals, but typically occurring in the inflammatory phase. In the presence of prolonged inflammation, the forming ECM is damaged, angiogenesis decreases, and re-epithelization is inhibited [109]. This leads to chronic healing and subsequently pathologic scarring, which is described as either hypertrophic scarring or keloid formation.

### Hypertrophic Scarring 

Hypertrophic scars (HTS) are scars with excessive collagen deposits during the wound healing process which causes them to have a raised appearance relative to the adjacent normal skin, but otherwise has borders confined to the injured area without overextension—a key characteristic of this class of pathologic scar formation. These wounds typically mature after about 2 years and do not recur after their excision. The incidence of post-burn contractures is common, with some data suggesting it occurs in as many as 30% of patients. This range is likely due to increased occurrence with severity of injury, where deeper and larger burns have a higher incidence [110].

Histologically from initial healing to final remodeling, HTS contain a higher density of collagen, myofibroblast occurrence, and glycosaminoglycans (GAGs) relative to normal scars [111]. Interestingly, the composition of these portions of the HTS also differ. For instance, there is initially an overproduction of type III to type I collagen, although both are produced. This ratio normalizes as healing progress, settling to that of normal scarring at maturity—about 6:1, type I and type III, respectively, [112]. Although there is more collagen present than in normal skin, the bundles within HTS are thinner than normal skin and are organized parallel to the basement membrane without the reticular orientation present in normal skin [113]. Other local factors disrupted in the ECM include proteins that restore wound integrity, such as elastin, which has been shown to be absent for several years following severe burns with prolonged healing, likely contributing to pathologic scar formation [14].

Fibroblasts in hypertrophic scars reflect the activity of fibroblasts found in the deep, reticular dermis of intact, normal skin. This is characterized by an upregulated expression of pro-collagen mRNA, and elevated levels of known cytokines linked to fibrosis, such as TGF-ß. In addition to increased activity related to collagen synthesis and ECM production, there is a decreased expression of factors related to remodeling, collagen breakdown and resorption of the ECM. Fibroblasts in hypertrophic scars have reduced matrix metalloproteinase-1 (MMP-1) activity, decreasing the occurrence of collagen breakdown. Nitric oxide levels, a known vasodilatior, are also lower [114]. This likely leads to less cellular recruitment locally, given less ample blood flow. Proteoglycan activity, involved in the restoration and creation of normal collagen fibrils, is inhibited. This was specifically found through reduced production of decorin, a proteoglycan that also binds TGF-β—whose known profibrotic activity is therefore potentiated [115].

The location of injury is also relevant for pathologic scar formation, in that myofibroblast differentiation is up regulated in wounds where higher tension is experienced, which has been associated with an increased incidence of hypertrophic scar formation. TGF-β has also been associated with increased fibrocyte generation, migration, and differentiation into myofibroblasts, solidifying its ongoing role with hypertrophic scar formation through matrix deposition, fibrosis, as well as tissue contraction. Myofibroblasts largely factor into the morbidity of wound contraction due to hypertrophic scarring through increased microfilament bundles and alpha smooth muscle actin activity [116].

Inflammatory cell recruitment is also affected, with an emphasis on helper T-cells (CD4+). Sub-type CD4+ cell infiltrate, varying from types 1–3 of helper T cells, has been shown to lead to either pro-fibrotic or anti-fibrotic wound environments. Type 1 helper T cells produce IL-2, interferon-gamma (IFN-γ), and IL-12, which are linked to anti-fibrosis and increased collagenase activity. Extrapolating this change in the microenvironment suggests that increases in Type 1 subsets of helper T-cells aid with remodeling and collagen degradation, which could inhibit hypertrophic scar formation. On the contrary, types 2 and 3 helper T cells are associated with the production of IL-4, IL-5, IL-10 and TGF-β. There subsets are linked to pro-fibrotic phenotypes with a decrease in collagenase activity—an environment which would lead to a lack of effort of wound remodeling, collagen degradation, and likely a pathway to hypertrophic scarring [108,116]. Treatments aimed at antagonizing TGF-β activity with IFN-α have demonstrated variable outcomes in burns and hypertrophic scars locally. Systemic administration of IFN, as opposed to local injection, is also under exploration. IFN-α has not been consistently shown to aid with outcomes, and its use is still in evolution, requiring further investigation [117,118].

## 7. Mitigation of the Post-Burn Inflammatory Response

Deep and large burns are devastating injuries that have not only local effects, but also serious systemic consequences through a severe and prolonged inflammatory response. Inflammation is vital to successful burn wound healing, but finding an appropriate balance is critical. As burn injury resuscitation and care evolves, the role that inflammation plays should not be ignored. This section aims to inject some of the proposed therapeutics and resuscitation schemes into this discussion as they pertain to post-burn inflammation.

### 7.1. Burn Resuscitation Schemes

As mentioned previously, one consequence of a large, systemic inflammatory response to burn injury is the onset of vascular leak. Significant edema is initiated by several factors including vasodilation, fluid extravasation, and increased microvascular permeability which often accompany inflammation [119]. Therefore, inflammation can influence the necessary fluid resuscitation requirements, however the inverse is also true. The fluid resuscitation methodology used has the potential to either mitigate or exacerbate post-burn inflammation processes. Gurney et al. (2019) makes the case for returning to the World War II era standard of using plasma for post-burn fluid resuscitation citing possible protection of the glycocalyx during post-burn endotheliopathy thereby avoiding further extravasation of inflammatory cytokines and worsening of tissue edema [120]. Similarly, it has been shown that administration of crystalloids during the resuscitation phase is associated with higher concentrations of proinflammatory cytokines and in turn higher expression of adhesion molecules which promote leukocyte infiltration [121]. Beyond the type of fluid used, the volume of resuscitation is of paramount concern when managing inflammation and post-burn edema with the notable concern of fluid-creep. Recent research has focused on optimizing the initial amount of fluids needed and what solutions are the most appropriate for burn patients. Studies have shown that the amount of fluid given during the first 24 h should be slightly higher than that projected by the Parkland formula. Several different solutions, including colloids and crystalloids, have been tested as the initial resuscitation fluid and the studies have indicated that a balanced crystalloid is the most optimal. Today, the Parkland formula remains the most widely used treatment for initial fluid resuscitation in burn patients [122].

### 7.2. Strategies to Decrease Inflammation

Furthermore, the coagulation cascade and the inflammatory response to burns are highly integrated and possess the potential for aggravated, reciprocal activation of one another. In turn, targeting coagulopathy may lead to mitigation of an excessive inflammatory response. One such proposed mechanism is the use of antithrombin (AT), which has already been examined in patients with severe sepsis [123]. Of note is the significant decrease in AT levels of burn patients during the first 24 h of post-burn care and that such a shortage has been shown to correlate with multi-organ failure, making replacement of AT a potential therapeutic for burn patients [124].

Burns typically keep progressing, and the non-salvageable zone of coagulation keeps expanding [125]. Therefore, superficial partial-thickness burns can become more severe. Deep partial- or full-thickness burns increase both surface area and depth of the burn, which further leads to more severe systemic and local symptoms [126]. Multiple factors cause burn wound conversion, such as decreased tissue perfusion, coagulation of the surrounding microvasculature, severe inflammation, free oxygen radicals and cell death. Several studies have demonstrated that burn wound progression can be reduced using therapeutics that would increase local perfusion, decrease inflammation, or have anti-coagulation and anti-apoptotic properties [127]. To increase perfusion in the zone stasis, Battal et al. investigated the effect of TAK-044, an endothelin-A and -B antagonist, on burn wound progression in rats. The results showed that intravenous (IV) administration of the molecule immediately after burn injury increased local perfusion, which decreased edema and inflammation, and led to significantly better tissue survival [128]. Multiple studies have investigated the effects of anti-inflammatory reagents on the treatment of burns. Eski et al. (2012) used topical cerium nitrate (CE) to decrease local inflammation immediately after burn injury in rats. Their study showed that topical application of CE reduced inflammation and decreased burn wound progression compared to control burns [129]. In order to reduce coagulation and burn wound progression, Yuhua et al. (2012) studied the effects of Poloxamer-188 (P-188), a surfactant with an anticoagulatory effect, in a rat burn model. The results of P-188 administered IV every 24 h demonstrated that the treatment significantly decreased secondary burn conversion compared to the untreated control group [130]. Taira et al. (2009) studied the effects of rosiglitazone, a thiazolidinedione, as an apoptosis inhibitor in a rat burn model. Their results showed that oral application of a PPAR-γ-ligand (nuclear-hormone-receptor), significantly reduced secondary burn wound conversion [131].

Many other therapeutics targeting inflammation have also been explored including stem cells, steroids, and opioids. Although the mechanisms of action have not been fully explored or established, bone marrow derived stem cells have been shown to be effective at modulating post-burn inflammation [132]. The use of steroids, such as methylprednisolone, and non-steroidal anti-inflammatory drugs, such as ketorolac, have been proposed and shown to reduce inflammation, pain, and length of hospital stay in burn patients [133,134]. Similarly, topical morphine has been shown to delay onset of the early inflammatory phase while accelerating the proliferative phase [135,136]. These findings are supported by in earlier in vitro work demonstrating that opioids can stimulate keratinocyte migration [137]. Large-scale clinical trials evaluating opioid efficacy on wound healing have not yet been conducted, however their primary use as an analgesic has made them the mainstay for pain management in burn patients prompting the need for further investigation [138,139].

## 8. Discussion

Previous studies have demonstrated that research has led to development of novel treatment strategies in burn care [133]. The prevention of burn wound progression with novel therapeutics, alternative methods to conventional split-thickness skin grafting (STSG) to provide wound coverage, as well as advancements in infection control, fluid resuscitation and nutritional support, has increased the survival rate and enhanced healing outcomes following severe burn injury (Table 2) [5].

The most common complications of burns are infections, which can progress to systemic sepsis, ultimately leading to increased morbidity and mortality [140,141]. Current burn infection prevention and treatment consists of various topical antimicrobial products, including silver, iodine and honey that are commonly impregnated into dressings such as films, foams, hydrogels, hydrocolloids and alginates. Topical delivery of antimicrobials is preferred approach because it prevents burn infections enabling application directly to the wound regardless of vascular damage, which potentially avoids adverse systemic effects while still providing high concentration at the target site [142]. Systemic antibiotic treatment is limited due to poor tissue penetration due to deranged microcirculation, and adverse side effects such as nephropathy, neuropathy and gastrointestinal disturbances, therefore they should only be administered for a short period of time [143,144,145]. Common short-term use includes, before, during and after surgical interventions, especially in patients with major burns [146]. As another attempt to avoid antimicrobial resistance and adverse effects, nanoformulations of antimicrobial peptides have been evaluated in wound healing. Antimicrobial peptides are endogenously produced by the cells of the skin and immune system to fight off pathogens, and therapeutics based on their mechanism of action, mainly introducing holes and destabilizing bacterial membranes, have demonstrated impressive results [147]. Future research is needed to evaluate the efficacy of high-concentration antimicrobial peptide nanoformulations as controlled release therapeutics for infection control in large burns. Furthermore, it has been shown that the endogenous gaseous small molecule mediator, nitric oxide (NO), has antibacterial activity and is involved in the regulation of hemostasis, inflammation and blood vessel and epidermal permeability as well as regulation of cell proliferation and tissue remodeling [148,149,150,151]. NO is produced by endothelial cells and inflammatory cells under physiological conditions and several pathophysiological states. Supplementation of NO to the wound to control infection, promote angiogenesis and promote wound repair and regeneration has demonstrated encouraging results [151]. However, the highly environment-dependent Janus-faced behaviour of NO needs to be taken into account when designing and targeting NO-releasing therapeutics for wound healing [152].

As stated, burns cause, not only local injuries but also significant systemic insults everywhere in the body. Therefore, burn research has also focused on improving treatment modalities to systemic responses. Prolonged and severe systemic inflammatory response to major burns can lead to hypovolemia and hypoperfusion called burn shock. Increased capillary permeability results in massive intravascular volume deficits and, therefore immediate fluid resuscitation and close monitoring for adequate, but not excessive, I.V. fluids are needed to prevent death. Fluid resuscitation is concentrated on supporting the patient over the first 24 h of profound hypovolemia and cardiac dysfunction [153]. Cardiac suppression is multi-factorial, mechanistically relating to over stimulation from catecholamines with beta-adrenergic receptors with several downstream effects, as well as the pro-inflammatory cytokine TNF-α. Prolonged elevation of catecholamines have been shown to disrupt calcium hemostasis intracellularly, inhibiting optimal cardiac function [154]. TNF-α effects are well studied and has been shown to cause negative inotropy and induce cardiomyocyte apoptosis both independent of and related to nitric oxide [155]. Hypotension may ensue due to depressed cardiac activity and from several other concurrent factors previously described. However, another important mediator of vascular pressure includes NO. Elevation of NO levels by NO synthase 2 activity induces vasodilation which may result in subsequent hypotension [155].

Furthermore, a substantial hypermetabolic response follows severe burns that can last more than a year post-burn. This state is described as abnormally increased circulatory volume, profound metabolic, physiologic, catabolic and immune system derangements [15]. Nutritional support is needed during and after major burn injury to meet the metabolic requirements since failure to fulfill the increased energy and protein needs can lead to multi-organ dysfunction, enhanced susceptibility to infection, and death [156]. The challenge is that for severe burn patients, the amount of nutrition necessary to prevent severe catabolism is usually unbearable to take orally due to altered mental status, inhalation injuries that compromise pulmonary function or cause gastrointestinal dysfunction and feeding intolerance [157]. Advances in burn care have led to the development of pharmacological treatment modalities against the hypermetabolic response. For example, daily administration of anabolic steroids, such as oxandrolone (a testosterone analog), has been shown to improve muscle protein degradation and reduce weight loss in burn patients [158]. The most efficient anti-catabolic treatment has been propranolol, a beta-blocker. Long-term use has been shown to decrease heart rate, fatty infiltration of the liver, and the amount of insulin necessary to reduce elevated blood glucose levels post-burn [159]. However, the physiology of the hypermetabolic response is complicated and the optimal route, volume, and composition of the nutritional support is not fully understood. Therefore, nutritional treatments should be individualized, monitored, and adjusted throughout recovery [24].

## 9. Conclusions

Burns are the result of cellular destruction within the cutaneous and subcutaneous tissues due to a rapid transfer of energy of variable magnitude to the body. The effects of burns can extend beyond the local zones of injury. The prolonged and strong immune response in major burns causes multiple systemic effects, damaging blood vessels, the heart, lungs, kidneys, and other organs. Furthermore, prolonged inflammation at the burn site can cause excessive growth of granulation tissue and wound contraction, which ultimately leads to scarring which can be disfiguring, functionally restrictive and may require revisionary surgeries. Minor, superficial burns usually heal well following the steps of normal wound healing. Deep, major burns require surgical treatment to heal. A better understanding of burn pathophysiology and the body’s systemic response to severe burn injury has resulted in progressive developments in burn care.

## Figures and Tables

**Table 1:** Immune and regenerative cells and their roles and characteristics during burn injury. Abbreviations: GM-CSF, granulocyte-macrophage colony stimulating factor; EGF, epidermal growth factor; FGF, fibroblast growth factor; IFN, interferon; IL, interleukin; KGF, keratinocyte growth factor; MHC, major histocompatibility complex; MMP, matrix metalloproteinase; MSH, melanocyte stimulating factor; PDGF, platelet derived growth factor; RNS, reactive nitrogen species; ROS, reactive oxygen species; TLR, toll-like receptor; TNF, tumor necrosis factor; TGF, transforming growth factor; VEGF, vascular endothelial growth factor.

	Cell Type	Function	Recruited or Activated by	Releases	Targets
**Innate immune cells**	Neutrophils	Microbe Elimination	P-Selectin (cell migration)	ROS	Pathogens
		Extracellular Matrix (ECM) Clearance		Extruded nets of histones and DNA	Lymphocytes
		Recruitment of Additional Inflammatory Cells		MMPs	Macrophages
				Elastase	Dendritic Cells
				Collagenase	Endothelial Cells
				TNF-α/β	Epithelial Cells
				IL-1β, IL-6	
	Monocytes	Immune Function Regulation	TLR-2, TLR-4	IL-1β, IL-8	Damaged Matrix and Cellular Debris
		Tissue Repair		TNF-α	
	M1 macrophages	Phagocytosis	Growth factors, cytokines (such as ILs)	Prostaglandin E2	Microbes
				ROS/RNS	Necrotic Cells
				TNF-α	Activated Lymphocytes/Th1 Cells
				IL-1, IL-6, and IL-8	
	M2 macrophages			IL-1 Receptor antagonist	M1 Macrophages
				PDGF	Polarized Th2 Cells
				TGF-β1	
				FGF	
	Mast cells	Immune Cell Recruitment and Migration	Resident Dermal Cells	Histamine	Endothelial Cells
		Dampens Excessive Immune Responses		TNF-α/β	Nerve endings
		Fibroblast Recruitment via Proteases		Cytokines (IL-1, IL-3, IL-5, IL-6, and IL-8)	Smooth Muscle Cells
				Growth Factors (VEGF, FGF, and PDGF)	
				Prostaglandins	
				GM-CSF	
				MIP-1β	
	Natural killer cells	Bacterial Infection Control via Cytolytic Activity	Type I + III IFN	Type II IFN + IFNγ	Neutrophils
			IL-12	TNF-α	Macrophages
			GM-CSF	GM-CSF	
				Cytokines (IL-2, IL-13, and IL-17)	
	Dendritic cells	Pathogen Recognition	TLR-7 + TLR-9	Type I IFN	Pathogens
		Induces Early Inflammatory Response			T-Cells (Activation)
		Re-epithelialization			NK Cells (Activation)
		Keratinocyte Proliferation			Keratinocyte (Proliferation)
		Enhances Antimicrobial Function of NK Cells			
		Activate T-Cells			
**Adaptive immune cells**	Helper T-Cells	Augmentation of the Innate Immune System	Cells Presenting MHC-I + -II	IL-2, IL-4, IL-5, IL-10, IL-17	B-Cells
			Cytotoxic T-Cells	IFNγ	Cytotoxic T-Cells
					Macrophages
	Killer T-Cells	Direct Defense Against Foreign Antigens	Cells Presenting MHC-I + -II	IL-2	Pathogens
				IFNγ	
	Unconventional T-Cells (MAIT, iNKT, γδ)	Modulation of the inflammatory response	Nonpeptidic antigens	Cytokines (such as IL-17)	Pathogens
			MHC molecules	Chemokines	Dendritic cells
					αß T-Cells
					B-Cells
	B-Cells	Immunoglobulin/Antibody Production	IL-4, IL-5, IL-15	Immunoglobulins/Antibodies	Pathogens
**Regenerative cells**	Keratinocytes	Formation of epithelium	Growth Factors (KGF, EGF, TGFβ, VEGF, and FGF)	Membrane Proteins (Collagen IV + VII)	Neutrophils
		Restoration of Barrier Function	Cytokines (IL-1, IL-6, IL-8, and TNF-a)	Growth Factors (MSF, NGF, VEGF, GM-CSF)	Macrophages
		Hair Follicle and Sweat Gland Regeneration	Integrins	Cytokines (TNF-α + IL-1α/β)	Fibroblasts
		Promotes Remodeling and Angiogenesis	Keratins		Melanocytes
			MMPs		Endothelial Cells
	Epidermal Stem Cells	Promote tissue regeneration	IL-1 and TNF-α	Cytokeratins	Epithelial Cells
				Growth Factors (EGF, TGF-β, VEGF, IGF)	Fibroblasts
				Basement Membrane Proteins	Endothelial Cells
					Smooth Muscle Cells
					Innate immune cells
	Melanocytes	Barrier Function	Tyrosine	Melanin	Keratinocytes
		Pigmentation	α-MSH		
		Prevention of UV Damage to the Integument			
	Fibroblasts	Promote connective Tissue Formation andvdermal Remodeling	TGF-β	Collagen	Endothelial Cells
				Fibrillin	Epithelial Cells
				Elastin	Immune Cells
				MMPs	Adipocytes
				Growth Factors (FGF, TGF-β, KGF, GM-CSF)	

**Table 2:** Burn research has led to the development of novel treatment strategies in burn care. The prevention of burn wound progression with novel therapeutics, alternative methods to conventional split-thickness skin grafting (STSG) to provide wound coverage, as well as advancements in infection control, fluid resuscitation, pain management and nutritional support, has increased the survival rate and enhanced healing outcomes following severe burn injury.

Major Burn (>20% TBSA)	
Local Treatment	Systemic Treatment
	
**Prevention of burn conversion**	**Burn shock**
Topical treatment	Fluid resuscitation
Systemic pharmacological agents	
	
**Infection control**	**Hypermetabolic state**
Topical antimicrobials	Nutrition support
Systemic antibiotics	
	
**Removal of necrotic tissue**	**Sepsis**
Surgical debridement	Systemic antibiotics
Enzymatic debridement	
	
**Barrier function restoration**	**Pain control**
STSG	Opiates
Minced skin transplantation	NSAID
Cell therapy	

## Data Availability

Not applicable.
